# Short-Term Long Chain Omega3 Diet Protects from Neuroinflammatory Processes and Memory Impairment in Aged Mice

**DOI:** 10.1371/journal.pone.0036861

**Published:** 2012-05-25

**Authors:** Virginie F. Labrousse, Agnès Nadjar, Corinne Joffre, Laurence Costes, Agnès Aubert, Stéphane Grégoire, Lionel Bretillon, Sophie Layé

**Affiliations:** 1 Nutrition et Neurobiologie Intégrée, INRA 1286, Bordeaux, France; 2 Nutrition et Neurobiologie Intégrée, University of Bordeaux, Bordeaux, France; 3 Eye and Nutrition Research Group, CGA, INRA Université de Bourgogne, Dijon, France; Max Delbrueck Center for Molecular Medicine, Germany

## Abstract

Regular consumption of food enriched in omega3 polyunsaturated fatty acids (ω3 PUFAs) has been shown to reduce risk of cognitive decline in elderly, and possibly development of Alzheimer's disease. Docosahexaenoic acid (DHA) and eicosapentaenoic acid (EPA) are the most likely active components of ω3-rich PUFAs diets in the brain. We therefore hypothesized that exposing mice to a DHA and EPA enriched diet may reduce neuroinflammation and protect against memory impairment in aged mice. For this purpose, mice were exposed to a control diet throughout life and were further submitted to a diet enriched in EPA and DHA during 2 additional months. Cytokine expression together with a thorough analysis of astrocytes morphology assessed by a 3D reconstruction was measured in the hippocampus of young (3-month-old) and aged (22-month-old) mice. In addition, the effects of EPA and DHA on spatial memory and associated Fos activation in the hippocampus were assessed. We showed that a 2-month EPA/DHA treatment increased these long-chain ω3 PUFAs in the brain, prevented cytokines expression and astrocytes morphology changes in the hippocampus and restored spatial memory deficits and Fos-associated activation in the hippocampus of aged mice. Collectively, these data indicated that diet-induced accumulation of EPA and DHA in the brain protects against neuroinflammation and cognitive impairment linked to aging, further reinforcing the idea that increased EPA and DHA intake may provide protection to the brain of aged subjects.

## Introduction

A decline in memory and cognitive functions, likely to rely on hippocampal dysfunction, is considered to be a normal consequence of aging [1]. Memory loss is a prominent health concern for older individuals (Alzheimer's Association health national survey, 2004). Some epidemiological studies suggest a role of long-chain ω3 polyunsaturated fatty acids (LCω3 PUFAs) in slowing cognitive decline in elderly individuals without dementia [2], [3], [4], [5].

Normal aging is also associated with increased brain inflammation characterized by elevated levels of proinflammatory factors such as proinflammatory cytokines interleukin (IL)-6, IL-1β and tumor necrosis factor α (TNFα) within the blood and the hippocampus [6], [7], [8], [9], [10], [11]. Proinflammatory cytokine expression is one attribute of glial reactivity, characterized by morphological and phenotypic modifications of microglia and astrocytes [12], [13], [14].

Neuroinflammation, which develops with age, may greatly contribute to cognitive impairment. Numerous studies have found a causal link between elevated cytokines levels in the brain and hippocampus-dependent memory deficits. Indeed, memory impairments induced by infection or stress are reversed by pharmacological inhibition of cytokine IL-1β [15], [16]. The detrimental role of IL-1β in learning and memory processes is reinforced by results showing that peripheral as well as intracerebral IL-1β injection impairs long-term memory [17], [18]. IL-6-deficient mice develop less memory impairments and display an attenuated induction of proinflammatory cytokines in the pyramidal cell layer of the hippocampus in response to a bacterial endotoxin as compared to wild-type mice [19]. In addition, in adult and aged rodents, these cytokines alter long-term potentiation (LTP), a cellular model of synaptic plasticity that has often been argued to play a role in learning and memory [20], [21], [22]. Very recently, study on the temporal relationship between cognitive aging and molecular changes in the CA1 region has revealed that neuroinflammatory processes are relevant candidates for triggering age-related cognitive decline [23]. Interestingly, inhibition of microglial activation and cytokines production by minocycline restores hippocampal LTP in aged rats [24] and improves memory in an animal model of Alzheimer's disease [25]. All together, these findings support the idea that neuroinflammation is a decisive component of cognitive disorders in aged subjects.

Thus, neuroinflammation is a good target to limit development of cognitive deficits with age [26]. LCω3 PUFAs, docosahexaenoic acid (DHA) and eicosapentaenoic acid (EPA) have anti-inflammatory properties [27] Reinforcing these data, we have recently demonstrated that DHA potently blocked *in vitro* endotoxin-induced cytokines production by microglial cells [28] and that a decrease in its brain level by dietary means [29] potentiated endotoxin-induced IL-6 production in the mouse hippocampus [30]. Very recently, we demonstrated that lifelong consumption of a control diet well-balanced in ω3 and ω6 PUFAs (in form of α-linolenic acid, α-LNA and linoleic acid, LA) increased brain DHA level as compared to an α-LNA-deficient diet [10]. However, lifelong consumption of the control diet was not sufficient to protect aged mice from proinflammatory cytokines expression and spatial memory decline [10].Interestingly, dietary intake of DHA and EPA has been shown to be involved in pathways likely to influence cognitive processes in elderly subjects and aged rodents [31], [32], [33], [34], [35], [36], [37]. However its effect on age-related neuroinflammatory processes has been poorly evaluated.

In this study, we hypothesized that a supplementation with the LCω3 PUFAs (DHA and EPA) over a two-month period may protect the brain from an uncontrolled inflammatory response and consequent cognitive decline. We demonstrated here that a two-month supplementation with dietary sources of LCω3PUFAs improved spatial memory and prevented from age-related neuroinflammatory processes.

## Materials and Methods

### Animals and Diets

All animal experiments were conducted according to the INRA Quality Reference System, and to relevant French (Directive 87/148, Ministère de l'Agriculture et de la Pêche) and international (Directive 86/609, November 24th 1986, European Community) legislation. This study was approved by INRA. Protocols were in keeping with protocols approved by Région Aquitaine Veterinary Services (Direction Départementale de la Protection des Animaux, approval ID: A33-063-920). Every effort was made to minimize suffering and the number of animal used. All experiments were performed with C57Bl6/J mice obtained from Charles River (Arbresle, France). They were maintained under standard housing conditions on corn cob litter in a temperature- (23±1°C) and humidity-controlled (40%) animal room with a 12 h light/dark cycle (7:00–19:00), with ad libitum access to food and water. Mice were raised and housed at the laboratory in groups of three in transparent polycarbonate cages. Mice were given experimental diets ad libitum (pellets prepared by UPAE-INRA, Jouy-en-Josas, France replaced daily) as previously described [10], [29], [38], [39] (see Table 1 and 2). To protect fatty acids (FAs) from oxidation, 20 g/kg of DL-α-tocopheryl acetate was added to the diets (see table 1). The source of EPA and DHA is tuna oil [40]. Pellets were stored at +4°C and FA composition was regularly controlled via gas chromatography analyses of organic extracts from manufactured food pellets. After mating, C57BL/6J female mice were submitted to an essential fatty acid (EFA) diet containing a mixture of rapeseed oil (rich in α-LNA), high-oleic sunflower oil and palm oil resulting in an α-LNA/LA ratio of ¼ (control diet) throughout gestation and lactation as previously described [10], [29], [30]. Male offspring were fed with the same diet as their dam until they were 1 month or 20 months old; thereafter, they were either maintained on the control diet or changed on an isocaloric LCω3 PUFA-supplemented diet containing a mixture of rapeseed oil, high-oleic sunflower oil, palm oil and tuna oil resulting in a 10% EPA and 7% DHA diet (LCω3 diet) for 2 additional months (for diet composition, see Table 1 and Table 2). This period of time was chosen on the basis of previous studies showing that a 2 to 3-month supplementation with a diet enriched in LCω3 increases DHA levels in neural tissues [41] and retina [40].

**Table 1:** Composition of the diets (g/kg diet).

Ingredient	Amount
Casein	180
Cornstarch	460
Sucrose	230
Cellulose	20
Fat^1^	50
Mineral mix^2^	50
Vitamin mix^3^	10

1 : for detailed composition, see Table 2.

2 : composition (g/kg): sucrose, 110.7; CaCO_3_, 240; K_2_HPO_4_, 215; CaHPO_4_, 215; MgSO_4_,7H_2_O, 100; NaCl, 60; MgO, 40; FeSO_4_,7H_2_O, 8; ZnSO_4_,7H_2_O, 7; MnSO_4_,H_2_O, 2; CuSO_4_,5H_2_O, 1; Na_2_SiO_7_,3H_2_O, 0.5; AlK(SO_4_)_2_,12H_2_O, 0.2; K_2_CrO_4_, 0.15; NaF, 0.1; NiSO_4_,6H_2_O, 0.1; H_2_BO_3_, O.1; CoSO_4_,7H_2_O, 0.05; KIO_3_, 0.04; (NH_4_)_6_Mo_7_O_24_,4H_2_O, 0.02; LiCl, 0.015; Na_2_SeO_3_, 0.015; NH_4_VO_3_, 0.01.

3 : composition (g/kg): sucrose, 549.45; retinyl acetate, 1; cholecalciferol, 0.25; DL-α-tocopheryl acetate, 20; phylloquinone, 0.1; thiamin HCl, 1; riboflavin, 1; nicotinic acid, 5; calcium pantothenate, 2.5; pyridoxine HCl, 1; biotin, 1; folic acid, 0.2; cyanobalamin, 2.5; choline HCl, 200; DL-methionin, 200; p-aminobenzoic acid, 5; inositol, 10.

**Table 2:** Fatty acid composition of the dietary lipids.

Diet	Control		LCω3	
	% wt of total fatty acids	g/kg diet	% wt of total fatty acids	g/kg diet
16:0	22.6	11.3	20.0	10.0
18:0	3.3	1.65	3.9	1.95
other saturated FAs	1.8	0.9	6.6	3.3
total saturated FAs	27.7	13.85	30.5	15.25
18:1ω9	57.9	28.95	22.6	11.3
18:1ω7	1.5	0.75	4.9	2.45
other monounsaturated FAs	0.6	0.3	6.7	3.35
total monounsaturated FAs	60.0	30.0	34.2	17.1
18:2 ω6 (LA)	10.7	5.35	15.2	7.6
18:3 ω3 (ALA)	1.6	0.8	0.9	0.45
20:5 ω3 (EPA)	n.d.	n.d.	10.9	5.45
22:5 ω3 (DPA)	n.d.	n.d.	1.1	0.55
22:6 ω3 (DHA)	n.d.	n.d.	7.2	3.6
Total ω3 PUFAs	1.6	0.8	20.1	10.05
total PUFAs	12.3	6.15	35.3	17.65
ω6/ω3	6.7	6.7	0.8	0.8

FAs, fatty acids; LA, linoleic acid; ALA, α-linolenic acid; EPA, eicosapentaenoic acid; DPA, docosapentaenoic acid; DHA, docosahexaenoic acid, PUFAs, polyunsaturated fatty acids.

A total of 54 mice divided in two cohorts were used for the following experiments. One cohort was used for behavioral measures (control diet: n = 7/young mice and n = 7/old mice; LCω3diet: n = 9/young mice and n = 7/old mice), c-Fos immunohistochemistry (6 mice were randomly chosen from each group) and morphometric analysis of astrocytes (3 mice were randomly chosen from each group). A second cohort (n = 6 per group)was used for the assessment of plasmatic cytokines by BioPlex, hippocampus-located cytokines by RT-PCR and cortical FAs levels.

### Behavioral measures

Behavioral testing took place in the morning (between 8:00 AM and 11:00 AM). Mice were handled for 5 min every day for two weeks prior the experiments. All the experiments were performed in an adjacent room to the animal facility under 78 lux of illumination.

Spatial recognition. Spontaneous spatial recognition in the Y-maze was used as a hippocampus-dependent test in a two-trial procedure as previously described [10], [42]. The apparatus used was a Y-shaped maze made of gray plastic. Each arm was 34 cm long, 8 cm wide and 14 cm high. Extra-maze visual cues were placed around the testing room and kept constant during the test. All arms of the maze being identical, discrimination between novelty and familiarity was only based on the different aspects of the testing room. The floor of the maze was covered with soiled sawdust coming from the home cages of several animals, and was mixed between sessions in order to eliminate olfactory cues. In the first trial, one arm of the Y-maze was closed with a guillotine door and mice were allowed to visit the two other arms for 5 min. After a 5-min inter-trial interval (ITI), mice were placed again in the start arm for the second trial and allowed to freely explore all three arms for 5 min. Start and closed arms were randomly assigned for each mouse. Preference for novelty was also assessed using a minimal ITI between acquisition and retrieval (less than 1 min). This ITI was employed to control for potential motivational deficits and to verify that all groups performed more visits to the novel arm when mnemonic demand was minimal.We compared the time spent exploring the novel and the familiar arm during the first 3 minutes of the second trial.

Object recognition. The test was performed as previously described [42]. A week before the acquisition phase (trial 1), mice were acclimatized to a 40×40 cm box made of white-coated plywood with 16 cm high walls for 15 min per day. The floor of the box was covered with corn cob litter which was mixed between trials in order to remove olfactory cues. During the 10 min of the trial 1, two identical plastic blocks with a particular shape and color were presented to the animal. At the end of the trial 1, the mouse was replaced in its home cage for a 1 h inter-trial interval (ITI) and one of the familiar blocks was replaced by a novel object with a different shape and color to test for memory retention. During the retrieval phase (trial 2), animals had free access to the familiar and novel blocks for 5 min. Object's exploration was defined as the mouse pointing its nose towards the object and/or touching it with the nose. Analysis was based on the time spent exploring the novel and the familiar objects during the 5 min of trial 2.

### Tissue processing

After transcardiac perfusion with PBS (pH 7.4), followed by 4% paraformaldehyde, brains were removed, post-fixed for 4 h, cryoprotected in 30% sucrose for 24 h, snap frozen in liquid nitrogen, and stored at −80°C before sectioning. Free-floating 30 µm coronal sections through the hippocampus (from -0.9 mm to -3.1 mm relative to bregma) were collected on a cryostat for immunohistochemistry.

### c-Fos immunohistochemistry and quantification

C-fos imaging was used as a neuronal activity reporter to map neurons activated in the hippocampus after the memory test [43], [44]. In aged mice submitted to memory tests, c-Fos marker decrease in the hippocampus is known to predict spatial memory impairment [45], [46]. To analyze cellular activation in the hippocampus of mice under study, we measured the expression of c-Fos by immunohistochemistry as previously described [42]. Mice were sacrificed by injecting a lethal dose of pentobarbital 90 min after the acquisition phase of the spatial recognition task (Y-maze group) or after a free exploration test (control group). This delay was chosen based on previous data showing that 90 min corresponds to the c-Fos protein expression peak in the hippocampus of mice subjected to spatial memory tests [42]. Mice in the Y-maze group were subjected to two 5 min tests in the Y-maze with a 5 min ITI, whereas control mice were allowed to freely explore a new cage in the same room and for the same amount of time as their spatially-trained counterparts (i.e. two 5 min periods with a 5 min ITI). This control condition allows assessment of activation resulting from exploratory behavior, sensorimotor stimulation, stress, and other factors not related to the spatial task. Thus, the difference between these two profiles, i.e. activation induced in the Y-maze group and in the control group, reflects learning-specific changes.

To analyze c-Fos expression, sections were incubated overnight at room temperature (RT) in rabbit polyclonal antiserum raised against c-Fos (Santa Cruz Biotechnology, Santa Cruz, CA), diluted 1∶1000 in Tris Buffered Saline (TBS) containing 0.3% Triton X-100, 2% donkey serum and 1% bovine serum albumin (BSA), before being incubated for 2 h with biotinylated donkey anti-rabbit antibody (1∶1000; Amersham Pharmacia Biotech Europe, Freiburg, Germany). Sections were then incubated for 2 h with avidin-biotin peroxidase complex (1∶1000; Vectastain ABC kit, Vector laboratories, Burlingame, CA), and labeling was revealed with diaminobenzidine using the nickel-enhanced glucose oxidase method [47]. Sections were then mounted onto gelatin-coated glass slides. Negative controls in which the primary antibody was omitted did not show any immunolabeling.

Brain sections were examined under a light microscope with a 10X objective (Nikon Eclipse E 400, Nikon Corporation, Champigny-sur-Marne, France) and images were captured by a high-resolution digital Nikon DXM 1200 camera (Nikon Corporation). Camera aperture, magnification, light power, and exposure time were fixed for all images. Images were generated and saved using ACT-1 software (Nikon Corporation, Champigny-sur-Marne, France). Quantification of c-Fos immunoreactive cells was performed with the NIH-imaging software Scion Image (Frederick, MD). The mean number of c-Fos positive cells was quantified in all consecutive sections containing the hippocampus (5–6 sections per mouse) in both hemispheres.

### GFAP immunostaining, 3D-reconstruction and morphometric analysis of astrocytes

To analyze astrocyte morphology, fluorescent labeling for GFAP was carried out in the hippocampus as previously described [48] [38]. Sections were incubated overnight at RT with a rabbit anti-cow GFAP antibody (1∶1000) in PBS containing 3% goat serum (Sigma Aldrich Corporation, Saint Louis, MI) and 0.3% Triton X-100. After rinsing, sections were incubated for 2 h with Alexa 594-conjugated goat anti-rabbit antibody (1∶1000; Molecular probes, Eugene, OR). Sections were then rinsed in PBS, mounted and coverslipped withVectashield mounting medium (Abcys, Paris, France). A series of images with an interval of 0.5 µm in the z-axis was captured with a confocal microscope (Leica DMR upright TCS SP2 AOBS) equipped with a HeNe laser, a 63X oil-immersion lens and a 3.10X zoom in the dentate gyrus (DG), CA1 and CA3 of the median hippocampus. For each region, 3 confocal images of GFAP fluorescence from each hemisphere were acquired randomly from 3 sections/mouse. Morphology of astrocytes was analyzed using the 3-D reconstruction software IMARIS. Processes length and diameter were measured using the “filament tracer” program (see Figure S1).

### BioPlex cytokine measures

Blood samples from mice were collected in EDTA-coated vials and centrifuged for 15 min at 3000 g at 4°C, aliquoted and stored at −80°C. Biorad bioplex kits were used for all assays (BioRad, France) as previously described [10]. All samples were run in duplicate and were assayed for IL-1β, IL-6 and TNFα according to the manufacturer's instructions. Results were expressed in pg/ml. The detection range was 0.35–5695.9 pg/ml.

### Real-time PCR

Mice were sacrificed by injection of a lethal dose of pentobarbital immediately after completion of behavioral testing, and transcardially perfused with Phosphate Buffered Saline (PBS). Hippocampi were quickly removed and frozen on dry ice. Two µg of total RNA obtained from each brain area were reverse transcribed with Moloney Murine Leukemia Virus reverse transcriptase (Invitrogen, Cergy-Pontoise, France). Quantitative PCR was then performed using the Applied Biosystems Assay-on- Demand Gene Expression Products protocol, as previously described [30]. Briefly, cDNAs for IL-6, IL-1β, TNFα, CD11b, GFAP and a housekeeping gene (β2-microglobulin) were amplified by PCR using an oligonucleotide probe with a 5′ fluorescent reporter dye (6-FAM) and a 3′ quencher dye (NFQ). Fluorescence was measured using an AB 7500 Real-Time PCR system (Applied Biosystems, Foster city, CA) and final quantification was carried out using the comparative threshold (Ct) method as previously described [30], [42]. For each experimental sample, difference between target gene and housekeeping gene Ct values (ΔCt) was used to normalize for differences in the amount of total nucleic acid added to each reaction and in the efficiency of the RT step. Values were then expressed as relative fold change (RFC) of the mean ΔCt value obtained for the group of control mice (free exploration behavior, see above) (calibrator ΔCt) by subtracting ΔCt for each experimental sample from the calibrator ΔCt ( = ΔΔCt). The amount of target gene (linear value) normalized to the housekeeping gene and relative to the calibrator was determined by 2^−ΔΔCt^.

### Analysis of fatty acid levels in brain lipids

Analysis of fatty-acid levels in brain lipids was carried out in the brain in order to minimize the number of animals (the same mice were used for the RT-PCR measurement of cytokines in the hippocampus). Lipids were extracted from the brain of 3- and 22-month old mice and fatty acids were transmethylated according to Morrison and Smith. Fatty acid methyl esters were analyzed on a Hewlett-Packard 5890 series II gas chromatograph equipped with a split/splitless injector, a flame ionization detector (Palo Alto, CA), and a CPSil88-silica capillary column (100 m×0.25 mm i.d., film thickness 0.20 µm, Varian, Les Ulis, France). The injector and the detector were maintained at 250°C and 280°C, respectively. Hydrogen was used as a carrier gas (inlet pressure 210 kPa). The oven temperature was fixed at 60°C for 1 min, increased to 85°C at a rate of 3°C/min and then to 190°C at a rate of 20°C/min, and maintained at this temperature for 65 min. Fatty acid methyl esters were identified by comparison with commercial standards.

### Statistical analysis

All data are expressed as the mean ± SEM. A three-way analysis of variance (ANOVA) with age (young vs. aged) and diet (control vs. LCω3) as between-subjects factors and arm or object (novel vs. familiar) as the within-subjects factor was performed for spatial or object recognition analyses. Specific comparisons between novel and familiar (arms or objects) were assessed by paired Student's t-tests. Specific comparisons between young and aged mice or control and LCω3 mice were assessed by unpaired Student's t-tests. PUFA brain levels, c-Fos quantification, RT-PCR measurements and astrocytic processes length were analyzed by two-way ANOVAs (age x diet). If a significant interaction was revealed, ANOVAs were followed by Fisher LSD post-hoc comparisons. Relationship between the number of c-Fos positive cells and the recognition score (novel object exploration/familiar+novel object exploration) was evaluated using Bravais-Pearson's correlation tests. For all results, a p-value<0.05 was considered as significant.

## Results

### 1) Short-term dietary supplementation with LCω3 PUFAs increases DHA and EPA levels in the brain of aged mice

We first investigated the impact of diets on LC PUFA levels in the brain of young and aged mice (Table 3). Arachidonic acid (AA) level was significantly increased according to age (F(1,13) = 38.27, p<0.001) and diet, with no significant effect of the interaction between age and diet. Conversely, DHA and EPA levels were significantly decreased with age (EPA: F(1,13) = 57.42, p<0.001 and DHA: F(1,13) = 19.44, p<0.001) and increased after consumption of fish oil (EPA: F(1,13) = 572.02, p<0.001 and DHA: F(1,13) = 362.34, p<0.001) with a significant age x diet interaction (EPA F(1,13) = 32.30; p = 0.0001 and DHA F(1,13) = 6.88; p<0.05). Post-hoc analysis further revealed a significant increase of EPA and DHA in the brain of aged mice fed with the fish oil diet as compared to those fed with the control diet (p<0.001). These changes resulted in significant differences in LCω6/LCω3ratio (age effect: F(1,13) = 39.71; p<0.001). In addition, delta 5 desaturase ratio (AA/dGLA) was significantly increased with age (F(1,13) = 98.10; p<0.001) and diet (F(1,13) = 24.76; p<0.001) with no significant diet x age interaction. AA is the precursor of proinflammatory lipidic derivates, while dGLA and EPA are precursors of anti-inflammatory lipidic derivates [49]. The ratio of (dGLA+EPA)/AA is significantly decreased with age (F(1,13) = 139.64; p<0.001) and increased with diet (F(1,13) = 66.00; p<0.001) with a significant age * diet interaction (F(1,13) = 8.73; p<0.05). Post hoc analysis further revealed that this ratio was significantly higher in aged mice fed with the LCω3 diet as compared to those fed with the control diet (p<0.05).

**Table 3:** Brain fatty acid composition.

	Young		Aged		Statistical effects		
	Control dietn = 4	LCω3 dietn = 4	Control dietn = 4	LCω3 dietn = 4	Diet effect	Age effect	Diet x Age
	% of total fatty acids						
16:0	14.3±0.28	19.5±0.35	14.0±0.80	18.6±0.20	<0.001	<0.05	NS
18:0	16.8±0.51	18.1±0.17	16.9±0.54	17.0±0.69	<0.05	NS	NS
Total saturated fatty acids	34.8±0.73	39.0±0.50	33.8±0.78	36.9±0.53	<0.001	<0.01	NS
16:1 ω7	0.5±0.01	0.6±0.02	0.5±0.05	0.7±0.06	<0.001	<0.05	NS
18:1 ω9	20.2±0.59	17.8±0.27	21.7±0.30	19.1±0.36	<0.001	<0.001	NS
18:1 ω7	4.5±0.03	3.5±0.03	3.8±0.04	3.4±0.02***	<0.001	<0.001	<0.001
Total monounsaturated fatty acids	34.1±0.93	25.3±0.45	34.5±1.72	27.1±0.43	<0.001	NS	NS
18:2 ω6	0.4±0.04	0.2±0.00	0.4±0.03	0.3±0.01**	<0.001	NS	<0.01
20:2 ω6	0.2±0.01	0.1±0.00	0.1±0.01	0.05±0.01***	<0.001	<0.001	<0.001
20:3 ω6 (dGLA)	0.5±0.02	0.4±0.01	0.4±0.02	0.3±0.02	<0.001	<0.001	NS
20:4 ω6 (AA)	5.5±0.24	5.8±0.16	6.2±0.32	6.6±0.23	<0.05	<0.001	NS
22:4 ω6	2.3±0.03	1.3±0.03	2.2±0.04	1.7±0.13***	<0.001	<0.001	<0.001
22:5 ω6	0.1±0.02	0.2±0.01	0.1±0.01	0.1±0.02	<0.01	<0.001	NS
Total ω6 fatty acids	9.2±0.29	8.0±0.18	9.5±0.36	9.2±0.35	<0.001	<0.001	<0.05
20:5 ω3 (EPA)	0.1±0.01	0.4±0.01	0.05±0.01	0.2±0.04***	<0.001	<0.001	<0.001
22:5 ω3	0.2±0.01	0.6±0.03	0.2+0.02	0.4±0.05***	<0.001	<0.001	<0.001
22:6 ω3 (DHA)	9.2±0.65	15.9±0.34	8.7±0.87	13.7±0.46***	<0.001	<0.001	<0.05
Total ω3fatty acids	9.5±0.66	16.9±0.36	8.9±0.89	14.3±0.53***	<0.001	<0.001	<0.05
Total ω6+ω3 fatty acids	18.6±0.94	24.9±0.38	18.4±1.22	23.5±0.53	<0.001	NS	NS
Total DMA	12.5±0.73	10.7±0.18	13.3±0.31	12.4±0.70	<0.001	<0.001	NS
ω6/ω3	0.97±0.04	0.48±0.02	1.07±0.07	0.64±0.04	<0.001	<0.001	NS
LC ω6/LC ω3	0.9±0.04	0.5±0.02	1.0±0.07	0.6±0.04	<0.001	<0.01	NS
AA/dGLA	11.6±0.84	14.2±0.68	17.1±1.33	20.5±1.65	<0.001	<0.001	NS
(dGLA+EPA)/AA	0.10±0.01	0.14±0.01	0.07±0.00	0.09±0.01*	<0.001	<0.001	0.01

dGLA: dihomo-gamma-linolenic acid (20:3 ω6); AA: arachidonic acid (20:4 ω6); EPA: eicosapentaenoic acid (20:5 ω3); DHA: docosahexaenoic acid; DMA: dimethyl acetal; LC ω6: long chain ω6 (20:2 ω6+20:3 ω6+20:4 ω6+22:4 ω6+22:5 ω6); LC ω3: long chain ω3 (20:5 ω3+22:5 ω3+22:6 ω3); NS: not significant.

*** p<0.001 as compared to aged control diet;

** p<0.01 as compared to aged control diet;

* p<0.05 as compared to aged control diet.

### 2) Spatial memory deficits in aged mice are reduced by a 2-month dietary supplementation with LCω3 PUFAs

**Figure 1:**
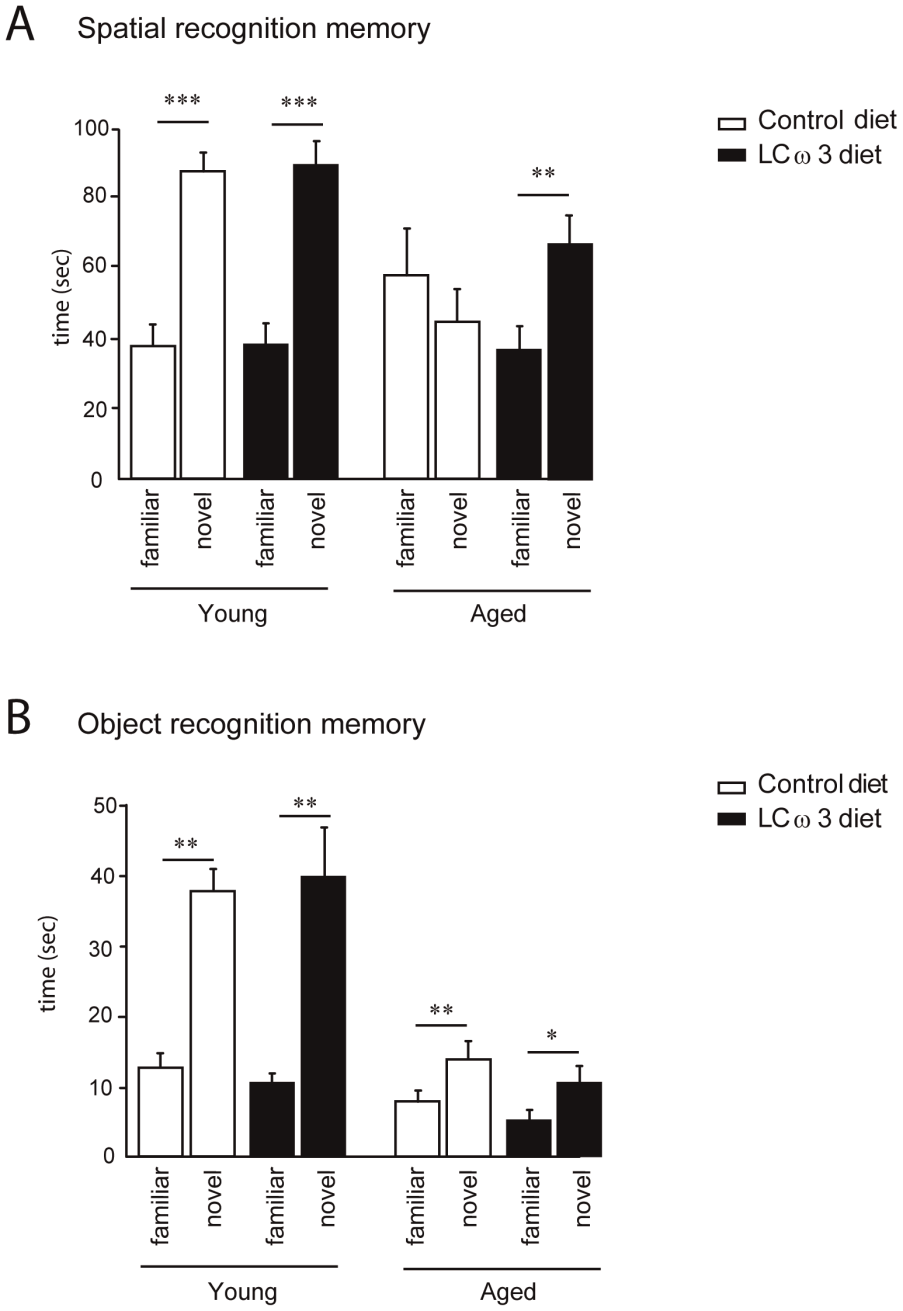
Spatial and object recognition memory. (A) Time spent (in sec) in the novel or the familiar arm after a 5-min ITI in 3-month-old (young) and 22-month-old (aged) mice fed with the control diet or the LCω3 diet for 2 months. (B) Time spent (in sec) in the novel or the familiar object after a 1-hr ITI in 3-month-old (young) and 22-month-old (aged) mice fed with the control diet or the LCω3 diet for 2 months. *** p<0.001, ** p<0.01, * p<0.05.

Spatial memory performance was assessed using a Y-maze test with a 5-min inter-trial interval (ITI) in adult and aged mice fed with the control or the LCω3 diet [10]. Spatial memory performances were significantly impaired with age and diet (Fig. 1A). A three-way ANOVA (age x diet x arm) revealed a significant effect of arm (F(1,52) = 31.7, p<0.001), and a significant interaction between age and arm (F(1,52) = 16.7, p<0.001) and diet and arm (F(1,52) = 5.7, p<0.05). Further analyses revealed that aged mice given the LCω3 diet significantly distinguished between the novel and the familiar arm (paired-t-test, t(8) = 4.8, p<0.01), unlike aged control mice, which exhibited a random exploration of the two arms (p = 0.55). We did not observed a difference in the time spent in the starting arm between aged mice fed with the control diet and those fed with the LCω3 diet (p = 0.159, data not shown). In order to evaluate whether the impairment revealed in aged mice fed with the control diet was a memory deficit or a performance deficit related to motivational alterations, animals underwent a spatial recognition test with minimal ITI between acquisition and retrieval. When mnemonic demand was minimal, all mice exhibited a clear preference for the novel arm (arm effect, F(1,52) = 135.6, p<0.0001; no effect of diet, F(1,52) = 0.003, n.s., or age, F(1,52) = 0.15, n.s., or interaction arm x diet, F(1,52) = 0.003, n.s., or arm x age F(1, 52) = 0.003, n.s.; data not shown).

Mice were submitted to the object recognition test which is based on the spontaneous tendency of rodents to spend more time exploring a novel object than a familiar one. This test is known to be hippocampus-independent [50]. A three-way ANOVA (age x diet x object) revealed a significant effect of object (F(1,52) = 52.2, p<0.001) and a significant interaction between age and object (F(1,52) = 21.9, p<0.001) (Fig. 1B). Interestingly, even if aged mice spent significantly less time exploring the new object as young mice did, all mice recognized the novel object compared to the familiar one, whatever the diet or the age of animals (paired-t-test: young control mice t(6) = 5.3, p<0.01; young LCω3 mice t(7) = 5.6, p<0.01; aged control mice t(9) = 4.5, p<0.01; aged LCω3 diet t(7) = 2.8,p<0.05).

**Figure 2:**
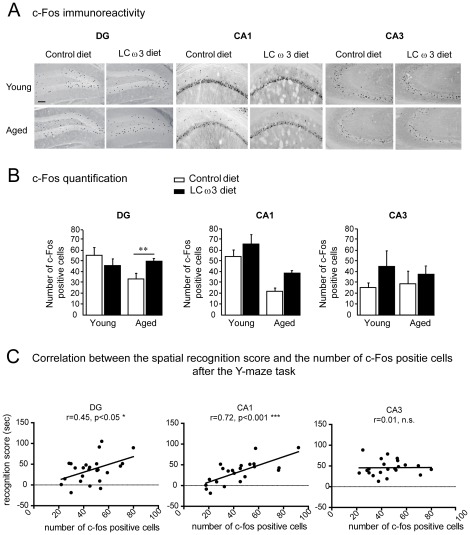
c-Fos expression in the DG, CA1 and CA3 regions of the hippocampus. c-Fos immunohistochemical analysis was performed in the hippocampus of 3-month-old (young) and 22-month-old (aged) mice fed with the control diet or the LCω3 diet for 2 months and sacrificed 90 min after the spatial recognition acquisition session. (A) Representative images of c-Fos immunohistochemistry in the DG (left panel), the CA1 (central panel) and the CA3 region (right panel) of the hippocampus. Scale bar = 100 µm. (B) Quantification of c-Fos-positive cells was performed in the DG, the CA1 and the CA3 regions of the hippocampus. Data are presented as mean ± SEM. ** p<0.01. (C) Correlation between the number of c-Fos positive cells induced by the Y-maze task in the DG (left panel), the CA1 (central panel) and the CA3 (right panel) and the spatial recognition score. Pearson's correlation coefficients (r) and corresponding significance (p) are displayed within each correlation window. The number of c-Fos positive cells in the DG and CA1, but not in the CA3 regions of the hippocampus was positively correlated to the spatial recognition score.

C-fos is expressed in an activity-dependent manner and has been used as a marker for activated neurons involved in learning and memory processes. In particular, we have previously shown that c-Fos is activated in the CA1 and DG, but not in the CA3 region of the hippocampus after a Y-maze task [42]. In addition, c-Fos activation is correlated with spatial memory performances in this task [42]. We therefore measured c-Fos expression in the DG, CA1 and CA3 regions of the hippocampus of young and aged mice fed with the control diet or the LCω3 diet after a Y-maze task (5 min ITI) or under control conditions (free exploration behavior) (Fig. 2A, 2B). In the CA1, CA3 and DG regions, no significant difference between age or diet was revealed under control conditions (free exploration behavior, an average of 8 to 11 c-Fos-positive cells per animal for DG and CA1 and an average of 30 c-Fos-positive cells for CA3 per animal, data not shown). After the Y-maze task, c-Fos was activated in the DG and CA1, but not in the CA3 (Fig. 2A, 2B) as previously described [42]. A two-way ANOVA did not reveal a significant effects of age (F(1,20) = 2.5, n.s.) or diet (F(1,20) = 0.37, n.s.), but revealed a significant interaction between age and diet (F(1,20) = 6.2, p<0.05) in the DG. Post-hoc analysis further revealed a significant increase of Y-maze-induced c-Fos positive cells in the DG region of aged mice (Fisher LSD, p<0.01) fed with the LCω3 diet as compared to control diet (Fig. 2A, 2B). However, the impact of Y-maze exposure differed across hippocampal regions. Indeed, a two-way ANOVA revealed an age effect (F(1,20) = 37.02, p<0.0001) and a diet effect (F(1,20) = 9.67, p<0.01) with no interaction (F(1,20) = 0.21, n.s.), in the CA1 region (Fig. 2A, 2B).

We further examined the relationship between c-Fos expression in the brain after the Y-maze task and spatial memory performances in this latter. As shown in Fig. 2C, c-Fos expression in the hippocampus was positively correlated to cognitive performances, in the DG and the CA1 (DG, r = 0.45, p<0.05; CA1, r = 0,72, p<0.001) but not in the CA3 (CA3: r = −0.01, n.s.).

### 3) Neuroinflammatory processes are reduced by a 2-month dietary supplementation with LCω3 PUFAs in the hippocampus of aged mice

**Figure 3:**
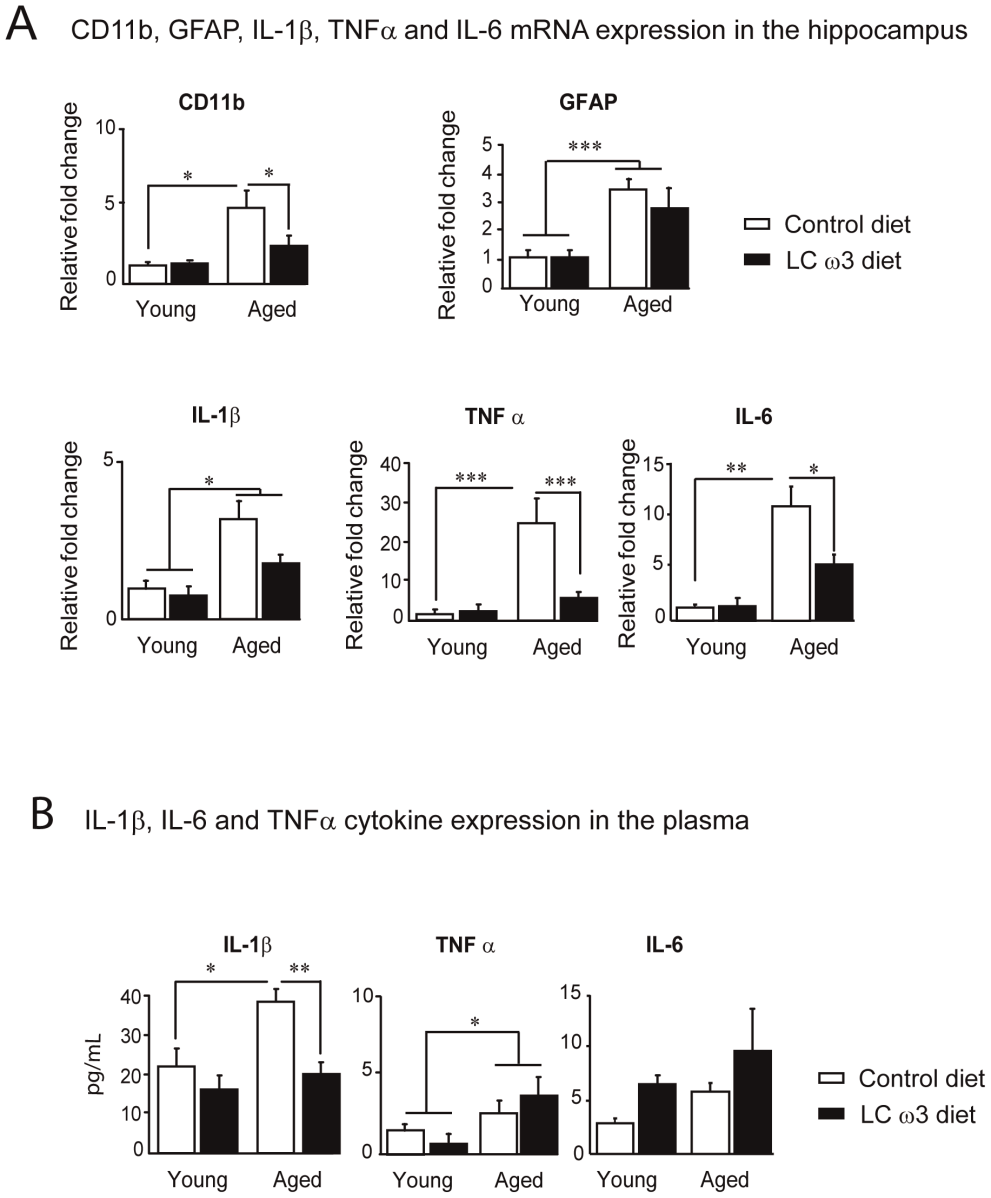
Cytokine expression in the hippocampus and in plasma. (A) CD11b, GFAP, IL-1β, IL-6 and TNFα mRNA expression was measured by real-time PCR in the hippocampus of 3-month-old (young) and 22-month-old (aged) mice fed with the control diet or the LCω3 diet for 2 months. Data are presented as mean relative fold change ± SEM. *** p<0.001, ** p<0.01, * p<0.05. (B) IL-1β, IL-6 and TNFα were measured by using a multiplex cytokines assays in the plasma. Data are presented as mean ± SEM in pg/ml. * p<0.05.

DHA potently reduces microglia-dependent activation and proinflammatory cytokines production in microglia [28]. Proinflammatory cytokines overproduction in the brain is linked to glial cells activation, in particular to microglia and astrocytes. One attribute of microglial activation is the overexpression of microglia-associated epitope CD11b [14]. With regard to astrocytes, the morphological alterations associated with aging are described as astrogliosis, a term that covers a rise in the expression of the astrocytic intermediate filament glial fibrillary acidic protein (GFAP). GFAP is a marker of astrogliosis that is elevated with age in mammals and is increased under inflammatory conditions [51]. Here, we investigated the effects of a short-term dietary supplementation with LCω3 PUFAs on CD11b, GFAP, IL-1β, IL-6 and TNFα mRNA expression in the hippocampus of aged mice. As shown in Fig. 3A, CD11b mRNA expression was significantly higher in the hippocampus of aged mice than in young mice (age effect: F(1,20) = 12.5, p<0.01; Fisher LSD, p<0.05). A LCω3 PUFA-supplemented diet significantly decreased mRNA levels of CD11b in aged mice (diet effect: F(1,20) = 4.9, p<0.05; Fisher LSD, p<0.05- interaction age x diet: F(1,20) = 4.47, p<0.05). GFAP mRNA expression increased with age (age effect: F(1,20) = 26.3, p<0.0001) but was not affected by diet (Fig. 3A). Results presented in Fig. 3A revealed a significant increase in TNFα, IL-6 and IL-1βmRNA expression throughout aging (age effect: F(1,20) = 85.2, p<0.0001; F(1,20) = 24.6, p<0.0001 and F(1,20) = 4.7, p<0.05 respectively). *Post-hoc* analyses further revealed that TNFα mRNA (Fisher LSD, p<0.001) and IL-6 mRNA (Fisher LSD, p<0.05) expression was significantly decreased to basal levels seen in young mice when aged mice were fed with the LCω3 diet, contrary to aged mice fed with the control diet (diet effect: F(1,20) = 47.6, p<0.001 for TNFα; F(1,20) = 5.3, p<0.05 for IL-6) (Fig. 3A).

IL-1β expression was significantly higher in the plasma of aged mice compared to young mice (age effect: F(1,15) = 8.7, p<0.01; Fisher LSD, p<0.05) (Fig. 3C). This was significantly reversed by a LCω3 PUFA-supplementation in aged mice (diet effect: F(1,15) = 12, p<0.01; Fisher LSD, p<0.01; interaction age x diet: F(1,15) = 3.5, p = 0.08). A two-way ANOVA revealed an increase in TNFα expression with age (age effect: F(1,15) = 5.3, p<0.05) whereas no effect of age or diet in IL-6 plasma levels was found in the four groups of mice (age effect: F(1,15) = 1.7, n.s.; diet effect: F(1,15) = 2.6, n.s.) (Fig. 3C).

In reactive astrocytes, GFAP labeling underlay an increase in the number, length and thickness of processes leaving the soma, leading to a hypertrophic phenotype [12], [13].

**Figure 4:**
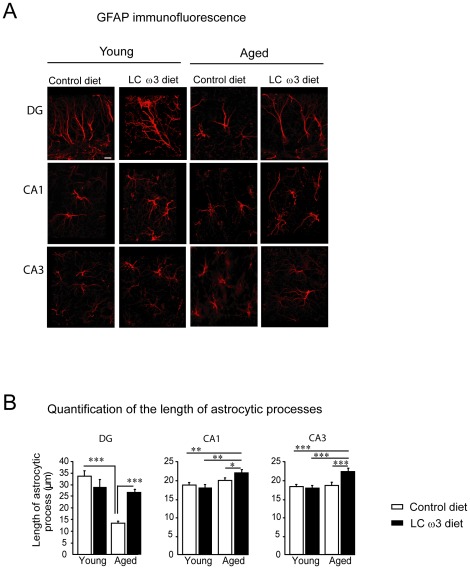
Morphometry of astrocytic processes in the hippocampus. (A) Confocal analysis of GFAP immunofluorescence was performed in the DG, the CA1 and the CA3 regions of the hippocampus of 3-month old (young) and 22-month old (aged) mice fed with the control diet or the LCω3 diet for 2 months using a 63X oil-immersion lens with a 3.10X zoom and IMARIS software. Images are representative of GFAP 3D immunofluorescence in the DG (upper panel), CA1 (central panel) and CA3 (lower panel). Scale bar = 8.5 µm. (A) Morphometric analysis of astrocytes in the DG, CA1 and CA3 regions of the hippocampus was performed using the “filament tracer” program/function. Data present means of primary and secondary processes lengths expressed in µm ± SEM. *** p<0.001, ** p<0.01, * p<0.05.

We then determined the effect of LCω3 PUFAs on astrocytes morphology in both young and aged mice. A quantitative morphometric analysis of GFAP-immunolabeled astrocytes has previously been used to demonstrate the occurrence of reactive astrocytes in the hippocampus of aged mice [52]. We analyzed astrocytic processes length by 3D-reconstruction analysis of GFAP immunofluorescence and subsequent quantification (Fig. 4A, 4B). In the DG of young mice, astrocytes had long processes extending from the hilus to the granule cell layer (f. 4A, left panel). In aged mice, astrocytes morphology was more “bushy-like” with shorter processes (Fig. 4A, central panel), as further revealed by quantification of astrocytic processes length (age effect: F(1,206) = 70.91, p<0.0001; Fisher LSD, p<0.0001) (Fig. 4B). With LCω3 PUFA supplementation, processes length in the DG of aged mice was partially restored (diet effect: F(1,206) = 5.88, p<0.05; age x diet interaction: F(1,206) = 57.34, p<0.0001 and Fisher LSD, p<0.0001) (Fig. 4A, right panel and 4B). Astrocytes had a more stellate shape in the CA1 and CA3 regions of both young and aged mice. A 2-month LCω3 PUFA supplementation had an effect on astrocytic processes length, as revealed by two-way ANOVA, in the CA1 (age effect: F(1, 180) = 9.43, p<0.01; age x diet interaction: F(1,180) = 4.34, p<0.05) and CA3 (age effect: F(1,192) = 9.94, p<0.01; diet effect: F(1,192) = 7, p<0.01; age x diet interaction: F(1,192) = 9.54, p<0.01). Post-hoc analysis further revealed that LCω3 PUFAs significantly increased the length of astrocytic processes in aged mice (Fisher LSD: young mice fed with control diet *vs.* aged mice fed with LCω3 diet, p<0.01 for CA1 and p<0.0001 for CA3; young mice *vs.* aged mice fed with LCω3 diet, p<0.01 for CA1 and p<0.0001 for CA3; aged mice fed with control diet vs. aged mice fed with LCω3 diet, p<0.05 for CA1 and p<0.001 for CA3) (Fig. 4A, 4B).

## Discussion

The work presented here provides direct *in vivo* evidence of anti-inflammatory and protective action of LCω3 PUFA supplementation on age-associated cognitive decline. Mice fed with the control diet, displayed poorer working memory performances with age. This was accompanied by an increase in the expression of CD11b and GFAP as well as mRNA levels of proinflammatory cytokines in the hippocampus. The increase in GFAP mRNA expression was associated with the appearance of shorter astrocytic processes in the DG, but not in the CA1 and CA3 as revealed by GFAP immunofluorescence. A 2-month dietary supplementation with fish oil in aged mice increased brain levels of DHA and significantly reduced mRNA levels of TNFα, IL-6 and CD11b. While GFAP expression was not reduced by the supplementation, the length of astrocytic processes in the DG was restored in the CA1 and CA3. In addition, working memory was improved by the LCω3 PUFA-supplemented diet. Taken together, our results provide new insight into the use of LCω3 PUFA supplementation in the elderly to limit development of neuroinflammation and cognitive impairment.

In the present study, we formulated 2 diets in order to provide different levels of DHA to the brain. In this work, the LCω3 PUFA supplementation was given for 2 months to mice fed throughout life with the control diet. This was of importance that the control and the LCω3 diets contained the same total amount of fat, therefore avoiding the possibility of a general effect of lipids. We previously showed that the long-term consumption of the control diet provided DHA to the brain of both young and aged mice [10], [29], [30]. We further found that adult and aged mice fed with the LCω3 PUFAs for 2 months had a higher DHA level in the brain compared to mice fed with the control diet. This is in agreement with previous studies showing that short-term consumption of a DHA-enriched diet in form of fish oil diet potently increased brain DHA level in young [53] and aged mice [31], [54], [55]. It is of importance that EPA was also increased in the brain of aged mice fed with LCω3 PUFAs, although the total levels remained very low as compared to those found for DHA. EPA increase has been recently reported in the brain of rats fed with a highly enriched EPA diet [56], however whether this increase was linked to preformed EPA incorporation in the brain or to a metabolic conversion of α-LNA remains to be determined.

Increased brain EPA and DHA levels in the aged brain were associated to the decrease in neuroinflammatory processes in the hippocampus. Particularly, age-associated increase of TNFα, IL-6 and CD11b mRNA expression was greatly reversed in the hippocampus. This was accompanied by the restoration of astrocytic phenotype in the DG region of aged mice. Addition of EPA and DHA to the diet could account for the improvement of neuroinflammatory processes linked to aging since these lipids have anti-inflammatory effects in the brain as shown by us and others [28], [53], [57], [58], [59], [60]. In our study, (GLA+EPA)/AA ratio was increased in the brain of aged mice fed with the fish oil diet, suggesting a balance in favor of anti-inflammatory prostaglandins production in the brain [57]. However, the role of anti-inflammatory prostaglandins remained to be determined. In addition, the delta 5 desaturase index increased suggesting that EPA did not limit synthesis of AA, as previously described in humans [61]. Indeed, the efficacy of EPA and DHA supplementation in decreasing proinflammatory cytokines production in the brain of aged mice could also be linked to other mediators such as resolvins or neuroprotectins [62], [63].

To our knowledge, this is the first evidence of the improvement of astrocyte morphology by a short term dietary LCω3 PUFA supplementation. Reactive astrocytes are normally characterized by the overexpression of GFAP, the major component of astrocytic cytoskeleton. Until recently, neuroinflammation-induced astrogliosis was described as a rise in the amount of GFAP and the development of a hypertrophic phenotype [12]. Our data confirmed the increase in GFAP expression already described in the aged human and rodent brain. More importantly, by using a 3D-reconstruction analysis, we showed a decrease in the length of astrocytic processes in the DG, but not in the CA1 and CA3 regions of aged mice. This is in agreement with another recent study, showing that the area occupied by GFAP-positive structures, as measured by an unbiased stereological approach, was reduced in the DG during aging, in spite of a better visibility of these structures due to increased GFAP content [64]. Morphological modifications of astrocytes in this context were dependent on proinflammatory cytokines. This change in the area occupied by astrocytes, rather than their number, has thus been suggested to better reflect functional changes in astrocytes [64]. While the increase in GFAP mRNA expression was not reversed by LCω3 PUFA supplementation in old mice in our study, the length of astrocytic processes in the DG was restored, accompanied by an increase in the astrocytic processes length in the CA1 and CA3 region of aged mice. In vitro, DHA influences astrocyte morphology and function rather than GFAP expression, corroborating our results [65], [66]. In addition, a beneficial role for DHA has been described in astrocyte maturation [66], which generally involves changing from a flat epithelioid morphology to a process-bearing stellate morphology [67]. Whether the effects of LCω3 PUFAs on astrocytes morphology in the hippocampus are direct or mediated by cytokines remains to be elucidated. However, knowing that LCω3 PUFAs have a strong impact on proinflammatory cytokines production in the brain [28], [30] and that proinflammatory cytokines, in particular IL-6, are produced by and activate astrocytes [68], [69], [70], it is tempting to speculate that the effects of LCω3 PUFAs on astrocytes morphology were linked to their effects on neuroinflammation.

Of importance, aged mice fed with the control diet displayed a decrease in spatial memory performances, as previously described [10]. In addition, c-Fos was activated in the CA1 and DG but not in the CA3 region of the hippocampus of young mice after the Y-maze task, as the hippocampus processes spatial information [42], [71], [72]. Interestingly, we found a decrease in c-Fos activation in the CA1 and DG regions of the hippocampus of old mice together with impaired Y-maze performances. The decreased expression of the activity-dependent immediate early genes (IEGs) c-Fos and Arc with age has already been reported, especially in the DG [73], [74], [75]. Given the role attributed to hippocampus and IEGs in spatial memory [76] and memory consolidation [43], it is likely that decreased c-Fos expression in the DG was a contributing factor in the age-related decline in hippocampal functions [77]. This was reinforced by the observation that alteration of the intrinsic properties or connectivity of the DG (e.g. insults or genetic variability) has previously been related to learning and memory deficits [78], [79], [80], [81], [82].

Several papers have reported the benefits of DHA and/or EPA on behavior, in particular in aged rodents [55], [83], [84], [85], [86]. However, in most of these studies control mice were submitted to an ω3 PUFA deficient diet [55]. In our study, control mice were fed with a control diet with a α-LNA/LA ratio of ¼. Very importantly, short-term LCω3 PUFA supplementation did not provide any cognitive benefits to young mice, but improved spatial memory with a short retention time in old mice. These results suggested that short-term DHA and EPA supplementation provided cognitive protection against spatial memory deficits linked to age in mice fed with a diet containing EFA α-LNA and LA throughout life. This was in accordance with recent studies showing a positive effect of short-term dietary supplementation with DHA and/or EPA on memory performances in aged mice [34], [37], [87]. Conversely, other studies reported a lack of protective effects of DHA/EPA-enriched diets on aged-associated cognitive decline [31], [34], [88]. Such discrepancies could be linked to the length of the supplementation time and the efficacy of LC PUFA increase in brain cellular membranes. Few studies have addressed the question of the duration of DHA dietary supplementation necessary to achieve a beneficial effect on memory in aged rodents. This would be of high interest in the context of age-associated cognitive decline in humans, in which handling of dietary LCω3 PUFAs could offer an efficient strategy for sustaining cognitive functions.

Indeed, increased level of EPA and DHA in the brain of aged mice could account for the effects of fish oil supplementation on spatial memory improvement. However, the mechanisms by which LCω3 PUFAs reduced cognitive impairment in aged rodents is not fully understood. According to our results, dietary LC PUFAs decreased neuroinflammatory processes in the hippocampus of old rodents. This was in accordance with previous studies showing that dietary ω3 PUFAs increased the recovery of LTP while reducing microglial activation in old rodents [89], [90], [91]. Of related interest was the prominent role played by proinflammatory cytokines in inducing cognitive deficits in a model of cerebral microvascular amyloid protein deposition, emphasized by a study using minocycline, a tetracycline that inhibits microglial activation [92]. This anti-inflammatory drug significantly improved cognitive performances, along with a reduction in the number of activated microglia and in IL-6 levels. In addition, sulindac, an anti-inflammatory compound [93], as well as minocycline improved learning and memory in a mouse model of Alzheimer's disease [25]. Importantly, minocycline partially restores LTP and proinflammatory cytokines expression in the hippocampus of middle-aged rats [24]. Very recently, neuroinflammatory processes have been identified as key early events (occurring in midlife, together with age-related cognitive changes) strongly implicated in cognitive dysfunction linked to age [23]. This is in accordance with results obtained in the lab (unpublished data) and by others showing that inflammatory events first induce production of proinflammatory cytokines in the hippocampus, followed by impairments in spatial memory/learning [19].

In this context, one can wonder what would be the amount of LCω3 PUFAs a human would have to consume to approximate the supplementation given to mice in our study and its protective effects. Epidemiological and clinical studies have used plasma and erythrocyte membrane levels of LCω3 PUFAs as a surrogate marker linking LC PUFAs status in the brain to cognitive disease risks, but results remain contradictory [94], [95]. To our knowledge, the precise incorporation into the aged brain of LC PUFAs from diet is not fully described. Recently, a study using positron emission tomography reported that the brain incorporation rate of DHA in form of free fatty acids is of 4.6 mg/day/1,500 g in healthy human volunteers with no changes in patients suffering from Alzheimer's disease [96], [97]. Further studies are therefore needed to relate human brain rates of DHA incorporation to dietary PUFA intake in aged subjects.

In conclusion, short-term LCω3 PUFA supplementation in aged mice shut down low-grade neuroinflammation, restored astrocytes morphology specifically in the DG region of the hippocampus and improved hippocampus-dependent cognitive performance, strongly supporting the view that dietary supplementation with LCω3 PUFAs could be used as a new therapeutic approach to prevent or reduce age-linked cognitive impairment.

## Supporting Information

Figure S1 **Imaging, 3D reconstruction and morphometric analysis of astrocytes.** (A) A series of consecutive 2D sectional images with an interval of 0.5 µm in the z-axis was collected with a confocal microscope using a 63X oil objective lens with 3.10X zoom (Leica DMR upright TCS SP2 AOBS) and 3D reconstruction was performed using IMARIS 64 Bitplane software. Each astrocyte was then isolated using the crop 3D function, and image processing, i.e. filtering of the images (Gaussian filter), including the removal of background noise and thresholding, was carried out, leading to B. (C) Morphometric measurements were performed using the “filament tracer program” (Matlab algorithm) allowing us to isolate each process of the astrocyte (represented by different colors). (D) The length of an astrocytic process was defined as the distance between the nucleus and the tip of an extended process identified by GFAP immunostaining. A Matlab algorithm was then used to produce a 3D-reconstruction image and the mean diameter of each astrocytic process.
(TIF)
