# Supplementary figures and images for: Short-Term Long Chain Omega3 Diet Protects from Neuroinflammatory Processes and Memory Impairment in Aged Mice
Source: PLoS One. 2012 May 25;7(5):e36861. doi: 10.1371/journal.pone.0036861 (PMC3360741; doi:10.1371/journal.pone.0036861)

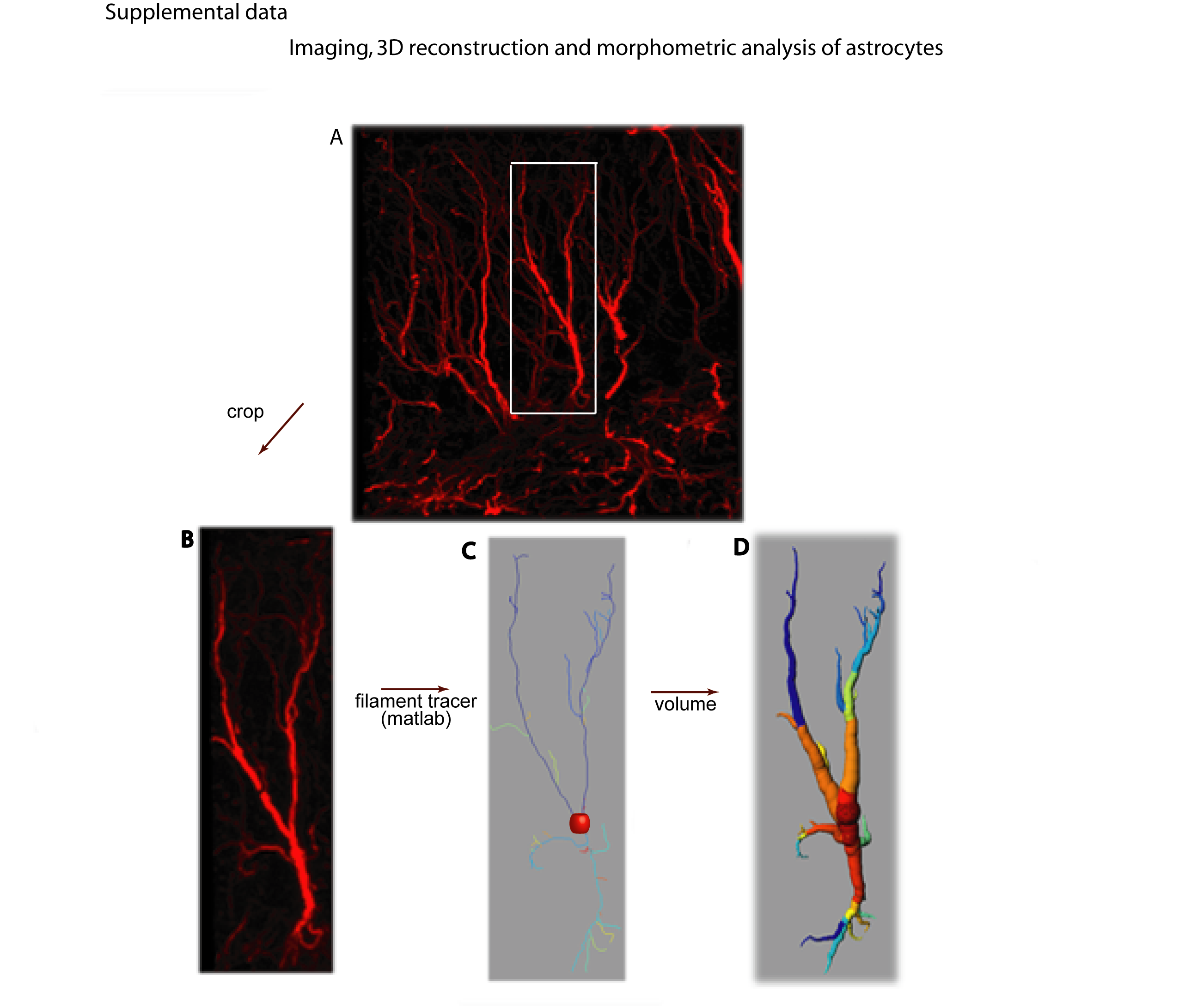

Supplement: Figure S1 — Imaging, 3D reconstruction and morphometric analysis of astrocytes. (A) A series of consecutive 2D sectional images with an interval of 0.5 µm in the z-axis was collected with a confocal microscope using a 63X oil objective lens with 3.10X zoom (Leica DMR upright TCS SP2 AOBS) and 3D reconstruction was performed using IMARIS 64 Bitplane software. Each astrocyte was then isolated using the crop 3D function, and image processing, i.e. filtering of the images (Gaussian filter), including the removal of background noise and thresholding, was carried out, leading to B. (C) Morphometric measurements were performed using the “filament tracer program” (Matlab algorithm) allowing us to isolate each process of the astrocyte (represented by different colors). (D) The length of an astrocytic process was defined as the distance between the nucleus and the tip of an extended process identified by GFAP immunostaining. A Matlab algorithm was then used to produce a 3D-reconstruction image and the mean diameter of each astrocytic process. (TIF) [file pone.0036861.s001.tif]
